# Supplementary material for: RIF1 regulates early replication timing in murine B cells
Source: Nat Commun. 2023 Dec 11;14:8049. doi: 10.1038/s41467-023-43778-y (PMC10713614; doi:10.1038/s41467-023-43778-y)
Supplement: Supplementary file 1 — Supplementary Information [file 41467_2023_43778_MOESM1_ESM.pdf]

SUPPLEMENTARY INFORMATION

RIF1 regulates early replication timing in murine B cells

Daniel Malzl[1,2,8], Mihaela Peycheva[1,3,8], Ali Rahjouei[4], Stefano Gnan[5], Kyle N. Klein[6], Mariia Nazarova[1], Ursula E. Schoeberl[1], David M. Gilbert[6], Sara C. B. Buonomo[5], Michela Di Virgilio[4], Tobias Neumann[1,7,9] and Rushad Pavri[1,9]

1. Research Institute of Molecular Pathology (IMP), Vienna Biocenter, 1030 Vienna, Austria
2. CeMM Research Center for Molecular Medicine of the Austrian Academy of Sciences, Lazarettgasse 14, 1090 Vienna, Austria
3. Present address: CeMM Research Center for Molecular Medicine of the Austrian Academy of Sciences, Lazarettgasse 14, 1090 Vienna, Austria
4. Max-Delbruck Center for Molecular Medicine in the Helmholtz Association (MDC), 13125 Berlin, Germany
5. Institute of Cell Biology, University of Edinburgh, Edinburgh EH9 3FF, UK
6. San Diego Biomedical Research Institute, San Diego CA 92121, USA
7. Present address: Quantro Therapeutics, Vienna Biocenter, 1030 Vienna, Austria.
8. Equal contribution
9. Corresponding authors (tobias.neumann@imp.ac.at, rushad.pavri@imp.ac.at)

Supplementary Figure 1

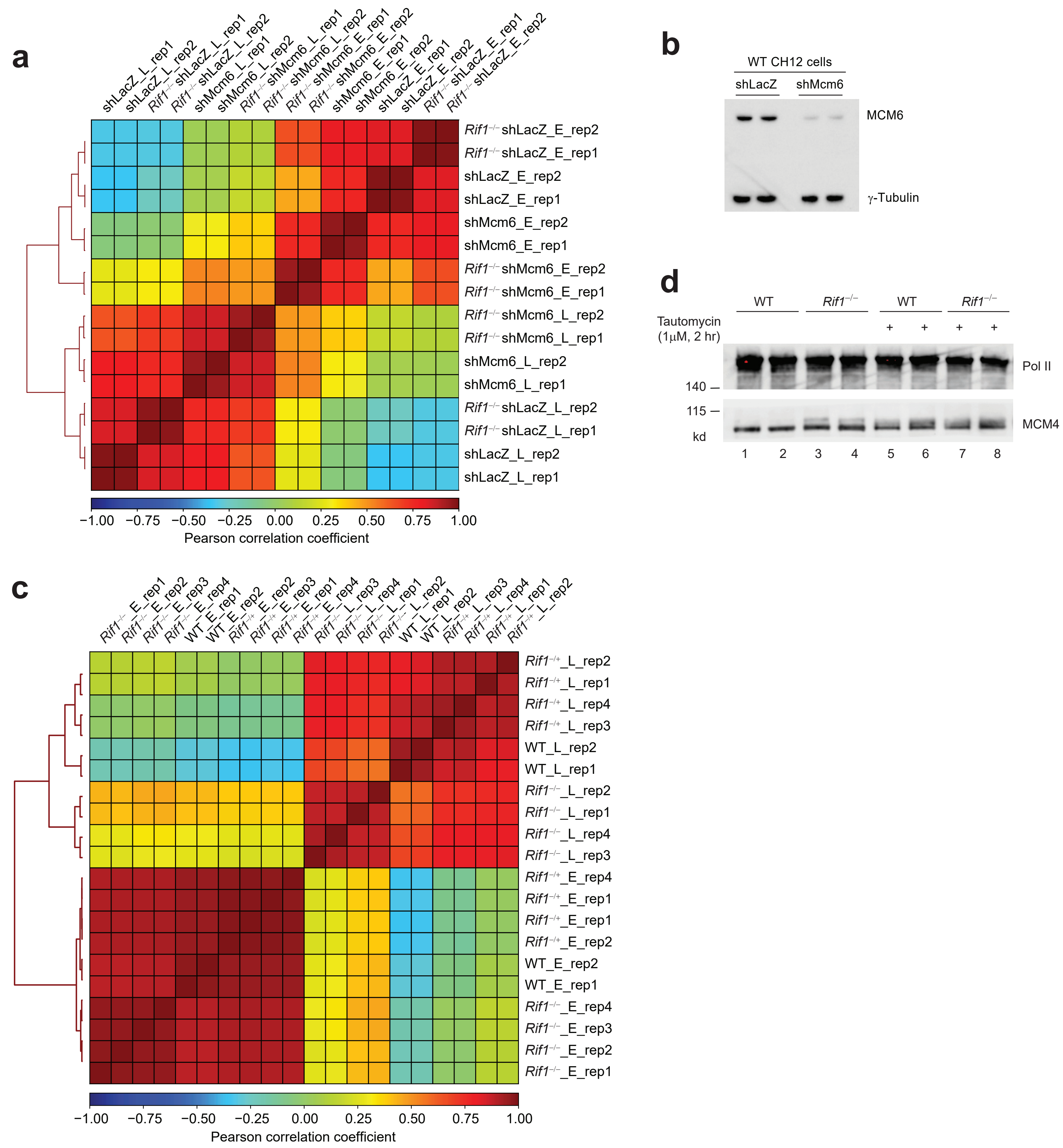

Supplementary Fig. 1 | Analysis of Repli-seq replicates. a, Pearson correlation matrix of Repli-seq data from two replicates (rep1 and rep2) of shLacZ, shMcm6, *Rif1*$^{-/-}$shLacZ and *Rif1*$^{-/-}$ shMcm6 CH12 cells. E and L indicate the early and late fractions, respectively. The color gradient (bottom bar) corresponds to the Pearson correlation coefficient. **b,** Western blot for MCM6 protein in nuclear extracts from shLacZ and shMcm6 cells. The experiment was performed twice and results from both experiments are shown with γ-Tubulin serving as the loading control. In all cases, 20 μg of extract was loaded. Source data are provided as a Source Data file. **c,** As in **a** from WT (two replicates), *Rif1*$^{-/-}$ (four replicates) and *Rif1*$^{-/-}$ (four replicates) primary, activated splenic B cells. **d,** MCM4 phosphorylation in chromatin fractions of WT and *Rif1*$^{-/-}$ CH12 cells ± 1μM tautomycin for 2 hr. The experiment was performed twice and results from both experiments are shown. Pol II is used as loading control. All lanes contain 10 μg protein. Source data are provided as a Source Data file.

Supplementary Figure 2

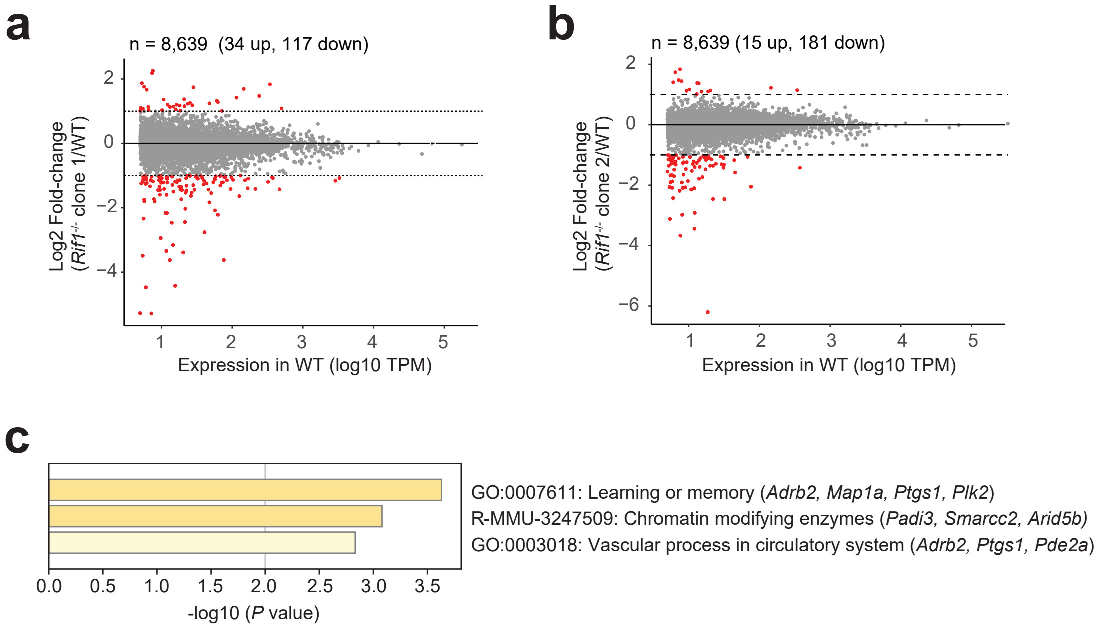

Supplementary Fig. 2 | RNA-seq analysis from *Rif1*$^{-/-}$ CH12 cells. a, RNA-seq analysis of *Rif1*$^{-/-}$ clone 1 compared to WT cells. The data are shown as a scatter plot of RNA-seq read densities in transcripts per million (TPM) versus fold-change (FC; *Rif1*$^{-/-}$/WT) for 8,639 expressed genes (defined as TPM > 5 in WT cells). The dotted lines mark log2 FC 1 or -1 corresponding to 2-fold upregulated and 2-fold downregulated genes, respectively. See also Supplementary Data 1. **b,** As in **a** but for *Rif1*$^{-/-}$ clone 2 compared to WT cells. **c,** GO term analysis of the downregulated genes common to *Rif1*$^{-/-}$ clones 1 and 2 (n = 22). There were no enrichments for the common upregulated genes (n = 3). See also Supplementary Data 1.

Supplementary Figure 3

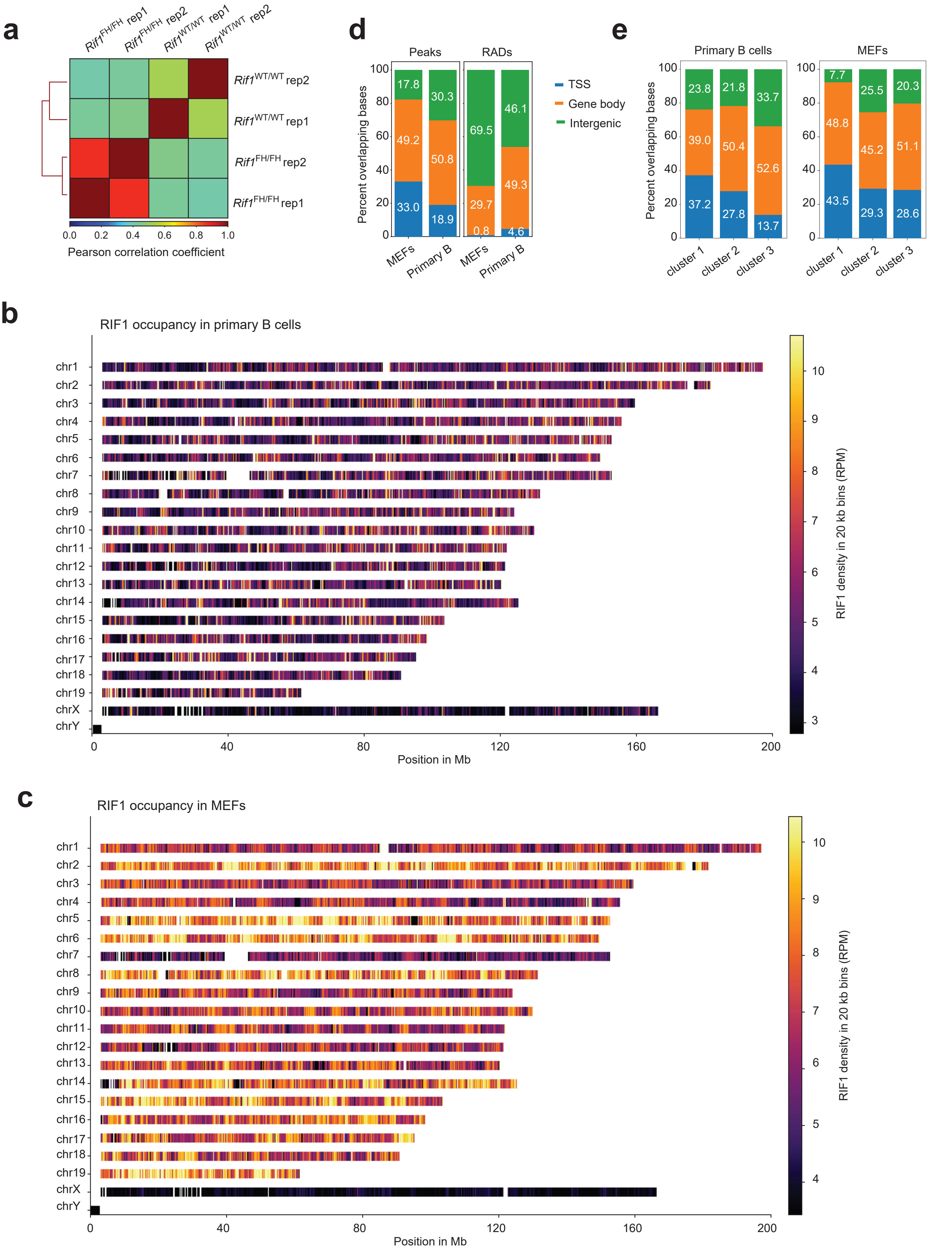

Supplementary Fig. 3 | Analysis of RIF1 ChIP-seq from primary B cells and MEFs. a, Pearson correlation analysis of RIF1 ChIP-seq datasets. Two replicates from *Rif1*$^{FH/FH}$ and *Rif1*$^{WT/WT}$ primary B cells were analyzed. **b,** RIF1 density from ChIP-seq in primary B cells shown across all mouse chromosomes in 20 kb genomic bins. **c,** As in **b** but for RIF1 ChIP-seq data from MEFs. **d,** Distribution of RIF1 peaks and RADs at TSSs (blue), gene bodies (orange) and intergenic regions (green). **e,** Distribution of RIF1 peaks from primary B cells (left) and MEFs (right) in TSSs (blue), gene bodies (orange) and intergenic regions (green) following k-means clustering described in Fig. 3e-f.

Supplementary Figure 4

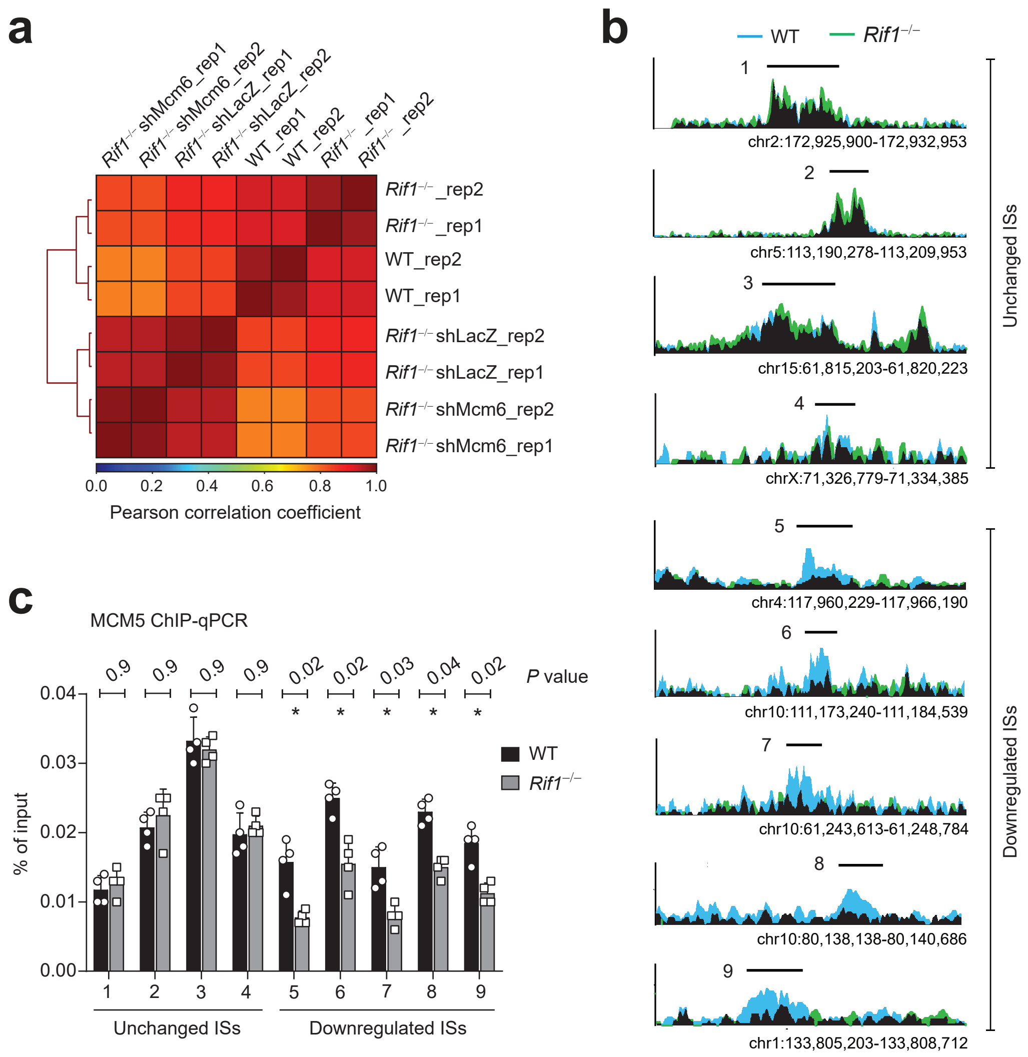

Supplementary Fig. 4: Correlation between SNS-seq replicates and analysis of RIF1 in origin licensing. a, Pearson correlation analysis of SNS-seq data from CH12 cells. Two replicates from each indicated condition were analyzed. **b,** Genomic snapshots of SNS-seq showing ISs that are unchanged (top four) or downregulated (bottom five) in *Rif1*$^{-/-}$ relative to WT CH12 cells. The tracks are overlaid with WT in blue, *Rif1*$^{-/-}$ in green and black being the overlap between them. The black bars indicate the ISs probed in **c** below. **c,** MCM5 ChIP-qPCR at the ISs highlighted in **b**. Data are from four experiments (N = 4) and represented as the mean ± standard deviation. Each sample is shown as a circle. Asterisks denote $P < 0.05$ via the unpaired Student's t test. Actual *P* value are shown at the top. Source data are provided as a Source Data file.

Supplemental Figure 5

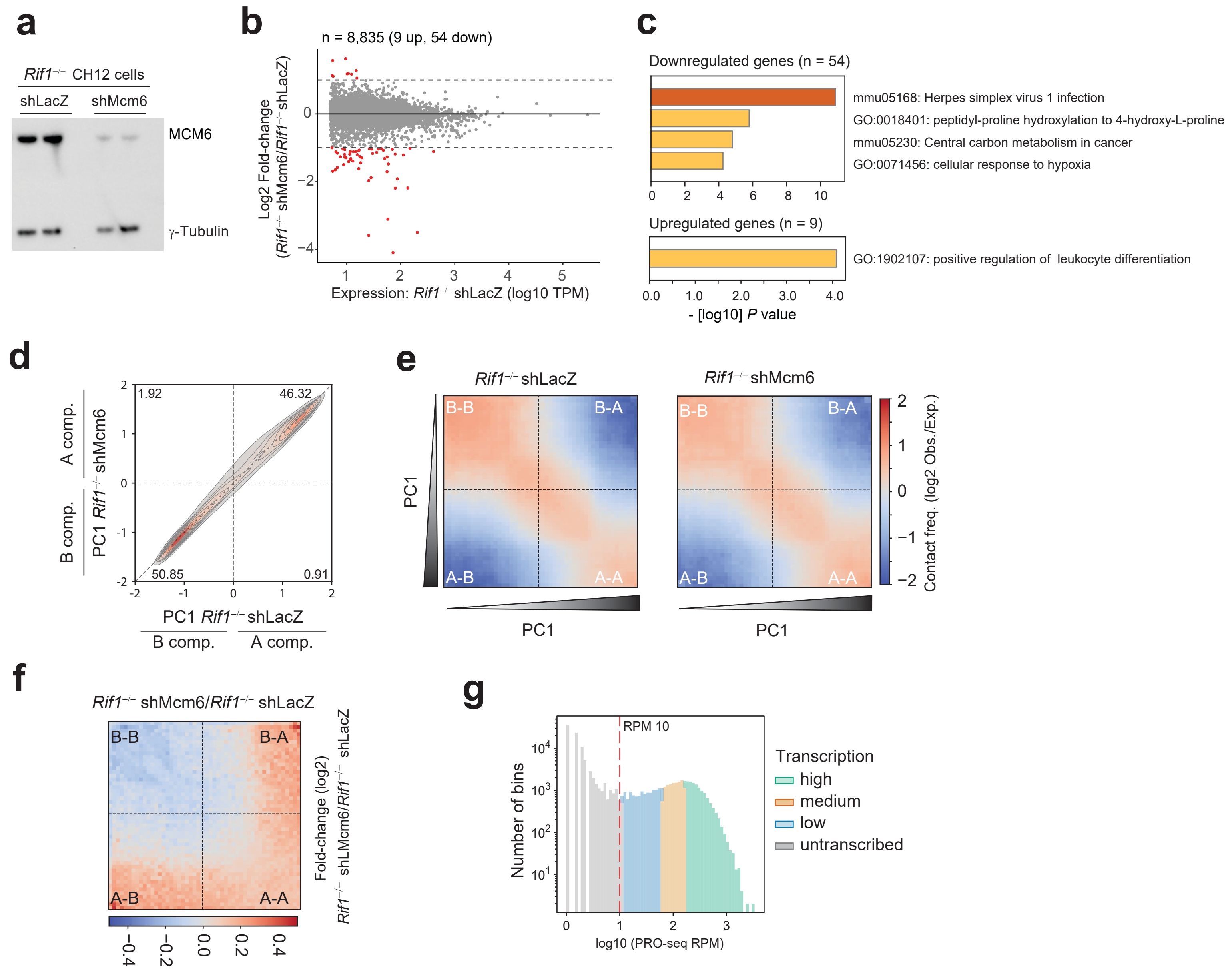

Supplemental Fig. 5 | Gene expression and genome compartmentalization analyses in *Rif1*$^{-/-}$ shMcm6 CH12 cells.

a, Western blot for MCM6 protein levels from nuclear extracts of *Rif1*$^{-/-}$ shLacZ and *Rif1*$^{-/-}$ shMcm6 CH12 cells. The experiment was performed twice and results of both are shown. In all cases, 20 μg of extract was loaded. γ-Tubulin serves as the loading control. Source data are provided as a Source Data file. **b,** Scatter plot of RNA-seq read densities in TPM versus fold-change (*Rif1*$^{-/-}$ shMcm6/Rif1*Rif1*$^{-/-}$ shLacZ) at 8,835 expressed genes (defined as TPM > 5 in *Rif1*$^{-/-}$ shLacZ cells). The dashed lines mark log2 FC 1 and -1 corresponding to 2-fold upregulated and 2-fold downregulated genes, respectively. **c,** GO term analysis of the 54 downregulated and 9 upregulated genes from **b**. **d,** Density-contour plot comparing PC1 compartment signals in 20 kb genomic bins from *Rif1*$^{-/-}$ shLacZ and *Rif1*$^{-/-}$ shMcm6 cells. **e,** Saddle plots comparing compartmental interactions between *Rif1*$^{-/-}$ shLacZ and *Rif1*$^{-/-}$ shMcm6 cells. **f,** Fold-change saddle plots based on data from **e**. **g,** Histogram showing the distribution of PRO-seq densities (RPM) in 20 kb genomic bins from shLacZ CH12 cells. High, Medium, Low and Untranscribed groups are color-coded to match the analysis in Fig. 7b. The dashed red line indicates RPM 10 which marks the boundary between transcribed and untranscribed regions in our analyses.
