# Supplementary material for: RIF1 regulates early replication timing in murine B cells
Source: Nat Commun. 2023 Dec 11;14:8049. doi: 10.1038/s41467-023-43778-y (PMC10713614; doi:10.1038/s41467-023-43778-y)
Supplement: Supplementary file 3 — Description of Additional Supplementary Files [file 41467_2023_43778_MOESM3_ESM.pdf]

Description of Additional Supplementary Files

File Name: Supplementary Data 1
Description: DESeq2 analysis of RNA-seq data from *Rif1*$^{+/+}$ and *Rif1*$^{-/-}$ CH12 cells. The data from each *Rif1*$^{-/-}$ clone is presented in separate tabs. Two additional tabs list the common 2-fold downregulated and 2-fold upregulated genes. A Wald test is used to estimate log2 fold-changes followed by a Benjamini-Hochberg multiple test correction.

File Name: Supplementary Data 2
Description: DESeq2 analysis of RNA-seq data from *Rif1*$^{-/-}$ shMcm6 cells relative to *Rif1*$^{-/-}$ shLacZ. A Wald test is used to estimate log2 fold-changes followed by a Benjamini-Hochberg multiple test correction.

File Name: Supplementary Data 3
Description: List antibodies and primers used in this study.

File Name: Supplementary Data 4
Description: Summary of data sets used in this study.

File Name: Supplementary Data 5
Description: Summary of software used in this study. All custom Python scripts and tools, except for scripts using ClusterScan, were developed, tested and executed using Python v3.7.3. ClusterScan and related scripts were executed using Python v2.7.13.
