# Supplementary material for: RIF1 regulates early replication timing in murine B cells
Source: Nat Commun. 2023 Dec 11;14:8049. doi: 10.1038/s41467-023-43778-y (PMC10713614; doi:10.1038/s41467-023-43778-y)
Supplement: Supplementary file 9 — Reporting Summary [file 41467_2023_43778_MOESM9_ESM.pdf]

nature portfolio

Corresponding author(s): Rushad Pavri and Tobias Neumann

Last updated by author(s): 2023/11/06

Reporting Summary

Nature Portfolio wishes to improve the reproducibility of the work that we publish. This form provides structure for consistency and transparency in reporting. For further information on Nature Portfolio policies, see our Editorial Policies and the Editorial Policy Checklist.

Statistics

For all statistical analyses, confirm that the following items are present in the figure legend, table legend, main text, or Methods section.

n/a	Confirmed	
☐	☒	The exact sample size (*n*) for each experimental group/condition, given as a discrete number and unit of measurement
☒	☐	A statement on whether measurements were taken from distinct samples or whether the same sample was measured repeatedly
☐	☒	The statistical test(s) used AND whether they are one- or two-sided<br>*Only common tests should be described solely by name; describe more complex techniques in the Methods section.*
☒	☐	A description of all covariates tested
☒	☐	A description of any assumptions or corrections, such as tests of normality and adjustment for multiple comparisons
☐	☒	A full description of the statistical parameters including central tendency (e.g. means) or other basic estimates (e.g. regression coefficient) AND variation (e.g. standard deviation) or associated estimates of uncertainty (e.g. confidence intervals)
☐	☒	For null hypothesis testing, the test statistic (e.g. *F*, *t*, *r*) with confidence intervals, effect sizes, degrees of freedom and *P* value noted<br>*Give P values as exact values whenever suitable.*
☐	☒	For Bayesian analysis, information on the choice of priors and Markov chain Monte Carlo settings
☒	☐	For hierarchical and complex designs, identification of the appropriate level for tests and full reporting of outcomes
☐	☒	Estimates of effect sizes (e.g. Cohen's *d*, Pearson's *r*), indicating how they were calculated

Our web collection on statistics for biologists contains articles on many of the points above.

Software and code

Policy information about availability of computer code

Data collection	NGS data was acquired using Illumina sequencing platforms or was downloaded from public repositories. Accessions are provided in the Supplementary Data 4.
Data analysis	If not stated otherwise, sequencing data was aligned against the mouse genome assembly of July 2017 (NCBI37/mm9). NGS track figures were generated with the UCSC genome browser. SNS-seq data was processed using the inisite-nf pipeline v0.1.0 [https://zenodo.org/doi/10.5281/zenodo.6827503] with trim_galore v0.6.4 for read quality control, Bowtie v1.2.3 for read alignment, MACS v2.2.6 for peak calling and clusterscan 0.2.1 and BedTools 2.29.2 for peak clustering, intersection and merging. RNA-seq data was trimmed with cutadapt v1.4.2 and filtered for rDNA contaminants using bowtie2 v2.1.0 (gi|374088139, gi|38176281). Filtered reads were aligned with STAR v2.4.2a and unique mappers were counted against the UCSC gene annotation (mm9 RefSeq) using featureCounts v2.0.0. Differential expression was analysed using DESeq2 v1.22.2 with subsequent log2 foldchange shrinkage using ashr v2.2-47. Repli-Seq data was processed with the repliseq-nf pipeline v0.1.0 [https://zenodo.org/doi/10.5281/zenodo.6823994] with trim_galore v0.6.5, bwa v0.7.17, picardtools v2.22.1, SAMtools v1.9, deepTools v2.5.1 [https://github.com/deeptools/deepTools] and BedTools v2.29.2. RT profile smoothing was performed with the loess function of R v3.5.1 and segmentation was performed with hmm_bigwigs v1.3 [https://github.com/gspracklin/hmm_bigwigs]. Hi-C data was processed with the hicer-nf pipeline v1.0.0 [https://doi.org/10.5281/zenodo.6868894] with trim_galore v0.6.5, HICUP v0.7.3, Bowtie2 v2.4.2, cooler v0.8.6 [https://github.com/open2c/cooler], krbalancing v0.0.5 [https://github.com/deeptools/Knight-Ruiz-Matrix-balancing-algorithm], pairix v0.3.7 and SAMtools v1.10. Principal components were computed with HOMER v4.1 and saddle plots and other analysis was performed with cooltools v0.3.2 [https://doi.org/10.5281/zenodo.3787004] and Python v3.7.3. PRO-seq data was processed using cutadapt v1.4.2, Bowtie v1.0.0, BedTools v2.27.1. Drosophila spike-in normalization was assessed by aligning against mm9/drosophila Flybase release 5 hybrid genome. ChIP-seq data was processed with trim_galore v0.6.4, Bowtie v1.0.0 and deepTools v3.3.0. Narrow peaks were called using MACS v2.2.6. Broad domains were called using EDD v1.1.19 [https://github.com/CollasLab/edd]. All additional analysis was done in Python v3.7.3

with pandas v1.1.0, pybedtools v0.8.0, numpy v1.16.4, matplotlib 3.1.0 and seaborn v0.11.1. A stable release of the analysis code can be found at https://doi.org/10.5281/zenodo.8183494. A table of all software used in provided in Supplementary Data 5.

For manuscripts utilizing custom algorithms or software that are central to the research but not yet described in published literature, software must be made available to editors and reviewers. We strongly encourage code deposition in a community repository (e.g. GitHub). See the Nature Portfolio guidelines for submitting code & software for further information.

Data

Policy information about availability of data

All manuscripts must include a data availability statement. This statement should provide the following information, where applicable:

- Accession codes, unique identifiers, or web links for publicly available datasets
- A description of any restrictions on data availability
- For clinical datasets or third party data, please ensure that the statement adheres to our policy

All next generation sequencing data (Repli-seq, RNA-seq, Hi-C, ChIP-seq and SNS-seq) has been deposited in GEO under accession number GSE228880 (https://www.ncbi.nlm.nih.gov/geo/query/acc.cgi?acc=GSE228880). The data processing workflows for the next-generation sequencing datasets with data analysis scripts are available centrally at https://github.com/pavrilab with stable version releases provided on Zenodo as follows: Repli-seq (https://doi.org/10.5281/zenodo.6823995), Hi-C (https://doi.org/10.5281/zenodo.6868894), SNS-seq (https://doi.org/10.5281/zenodo.6827504) and all other analyses (https://doi.org/10.5281/zenodo.8183494). Source data are provided with this paper.

Research involving human participants, their data, or biological material

Policy information about studies with human participants or human data. See also policy information about sex, gender (identity/presentation), and sexual orientation and race, ethnicity and racism.

Reporting on sex and gender	N/A
Reporting on race, ethnicity, or other socially relevant groupings	N/A
Population characteristics	N/A
Recruitment	N/A
Ethics oversight	N/A

Note that full information on the approval of the study protocol must also be provided in the manuscript.

Field-specific reporting

Please select the one below that is the best fit for your research. If you are not sure, read the appropriate sections before making your selection.

☒ Life sciences ☐ Behavioural & social sciences ☐ Ecological, evolutionary & environmental sciences

For a reference copy of the document with all sections, see nature.com/documents/nr-reporting-summary-flat.pdf

Life sciences study design

All studies must disclose on these points even when the disclosure is negative.

Sample size	Sample size was determined based on previous studies in the field (e.g.
Data exclusions	No data was excluded from the analyses.
Replication	Experiments were conducted at least twice in an independent manner. All attempts at replication were successful.
Randomization	Randomization was not relevant to the study. For mice, age-matched animals were used for experiments.
Blinding	Blinding was not performed during data collection and analysis because the investigators needed to verify genotypes and identify control and experimental conditions prior to starting the experiments.

Reporting for specific materials, systems and methods

We require information from authors about some types of materials, experimental systems and methods used in many studies. Here, indicate whether each material, system or method listed is relevant to your study. If you are not sure if a list item applies to your research, read the appropriate section before selecting a response.

Materials & experimental systems

n/a	Involved in the study
☐	☒ Antibodies
☐	☒ Eukaryotic cell lines
☒	☐ Palaeontology and archaeology
☐	☒ Animals and other organisms
☒	☐ Clinical data
☒	☐ Dual use research of concern
☒	☐ Plants

Methods

n/a	Involved in the study
☐	☒ ChIP-seq
☒	☐ Flow cytometry
☒	☐ MRI-based neuroimaging

Antibodies

Antibodies used	Anti-HA clone F-7 (Santa Cruz sc-7392), MCM6 clone H-8 (Santa Cruz sc-393618), beta-Tubulin clone TUB 2.1 (Sigma T-5201), MCM4 polyclonal (Abcam ab4459), RNA Pol II clone 4H8 (Abcam ab5408), MCM5 polyclonal (Bethyl Laboratories A300-195A).
Validation	Antibodies were used based on the manufacturer's validation and their use by other investigators (including our group) in previous publications. No additional validation was done. The website of the manufacturer may provide information on validation.<br>1. HA: https://www.scbt.com/p/ha-probe-antibody-f-7<br>2. MCM6https://www.scbt.com/p/mcm6-antibody-h-8<br>3. MCM4: https://www.abcam.com/en-at/products/primary-antibodies/mcm4-antibody-ab4459<br>4. MCM5: https://www.thermofisher.com/antibody/product/MCM5-Antibody-Polyclonal/A300-195A-T<br>5. RNA Pol II: https://www.abcam.com/en-at/products/primary-antibodies/rna-polymerase-ii-ctd-repeat-ysptsps-phospho-s5-antibody-4h8-chip-grade-ab5408<br>6. Beta-Tubulin: https://www.sigmaaldrich.com/AT/de/product/sigma/t5201

Eukaryotic cell lines

Policy information about cell lines and Sex and Gender in Research

Cell line source(s)	CH12-F3 cells (murine B cell lymhpoma line) obtained from Dr. Tasuku Honjo.
Authentication	These cells were originally derived in the lab of Dr. Tasuku Honjo and were authenticated in their original study (PMID: 8671604). No additional authentication was done.
Mycoplasma contamination	Cells were confirmed as Mycoplasma-negative.
Commonly misidentified lines<br>(See ICLAC register)	No commonly misidentified cell lines were used in the study

Animals and other research organisms

Policy information about studies involving animals; ARRIVE guidelines recommended for reporting animal research, and Sex and Gender in Research

Laboratory animals	C57BL/6 mice were used to obtain splenic B cells for primary cell cultures. Mice were 8-12 weeks of age. Mice were kept in a specific pathogen-free (SPF) barrier facility at a temperature of 20 ± 2°C, humidity at 55% ± 15% and a 12hr:12 hr light:dark cycle. Animals were maintained in small groups (4–5) or as breeding pairs in individually ventilated cages and had uninterrupted access to food and water.
Wild animals	The study did not use wild animals.
Reporting on sex	Both female and male mice were used at similar ratios in this study.
Field-collected samples	The study did not involve samples collected in the field.
Ethics oversight	All experiments were performed in compliance with the European Union (EU) 627 directive 2010/63/EU, and in agreement with Landesamt für Gesundheit und 628 Soziales directives (LAGeSo, Berlin, Germany).

Note that full information on the approval of the study protocol must also be provided in the manuscript.

Plants

Seed stocks	N/A
Novel plant genotypes	N/A

Authentication	N/A

ChIP-seq

Data deposition

☒ Confirm that both raw and final processed data have been deposited in a public database such as GEO.

☒ Confirm that you have deposited or provided access to graph files (e.g. BED files) for the called peaks.

Data access links<br>*May remain private before publication.*	The data is deposited in GEO under accession number GSE228880 (https://0-www-ncbi-nlm-nih-gov.brum.beds.ac.uk/geo/query/acc.cgi?acc=GSE228880).
Files in database submission	GSM7140982 priB_Rif1FH_ChIPseq_rep1<br>GSM7140983 priB_Rif1FH_ChIPseq_rep2<br>GSM7140984 priB_Rif1WT_ChIPseq_rep1<br>GSM7140985 priB_Rif1WT_ChIPseq_rep2
Genome browser session<br>(e.g. UCSC)	http://pavri-ucsc.imp.ac.at/natcommsession/tracks/hub.txt

Methodology

Replicates

Technical replicates were included in all experiments. In some cases, experiments were repeated independently to ensure reproducibility.

Sequencing depth

Sample	number of raw reads	uniquely aligned reads	read_length	is_paired
priB_Rif1FH_ChIPseq_rep1	31,644,997	25,379,657	100	No
priB_Rif1FH_ChIPseq_rep2	32,321,383	25,610,870	100	No
priB_Rif1WT_ChIPseq_rep1	39,245,096	29,142,151	100	No
priB_Rif1WT_ChIPseq_rep2	47,927,217	36,682,290	100	No

Antibodies	Anti-HA (Santa Cruz sc-7392)
Peak calling parameters	See the Methods section under Bioinformatics.
Data quality	Sequencing reads that passed Illumina quality controls were used for alignment and only uniquely aligned reads were used for all analyses.
Software	See the Methods section under Bioinformatics which provides tables with a details of the software used.
